# Fatty Liver Is Associated with Transcriptional Downregulation of Stearoyl-CoA Desaturase and Impaired Protein Dimerization

**DOI:** 10.1371/journal.pone.0076912

**Published:** 2013-09-30

**Authors:** Tomas Fernández Gianotti, Adriana Burgueño, Noelia Gonzales Mansilla, Carlos Jose Pirola, Silvia Sookoian

**Affiliations:** 1 Department of Molecular Genetics and Biology of Complex Diseases, Institute of Medical Research A Lanari-IDIM, University of Buenos Aires-National Council of Scientific and Technological Research (CONICET), Ciudad Autónoma de Buenos Aires, Argentina; 2 Department of Clinical and Molecular Hepatology, Institute of Medical Research A Lanari-IDIM, University of Buenos Aires-National Council of Scientific and Technological Research (CONICET), Ciudad Autónoma de Buenos Aires, Argentina; The University of Manchester, United Kingdom

## Abstract

**Aims and Methods:**

We evaluated the modulation of liver stearoyl-CoA desaturase-1 (Scd1) by dietary factors and insulin resistance (IR) in two experimental models of high-fat diet (HFD)-induced nonalcoholic fatty liver disease (NAFLD). The first model included Sprague Dawley (SD) rats that developed NAFLD without IR, and the second one included a rat model of genetic IR and cardiovascular disease, the spontaneously hypertensive rats (SHR) and its normotensive, insulin-sensitive control Wistar-Kyoto (WKY). The adult rats were given standard chow diet (CD) or HFD for 10 weeks. In all the animals, we explored the hepatic Scd1 transcriptional activity and protein levels.

**Results:**

HFD-fed rats of both strains developed severe NAFLD. Liver abundance of *Scd1* mRNA was significantly decreased in HFD-fed rats regardless of the strain; SD-CD: 235±195 vs. SD-HFD 4.5±2.9, p<0.0004, and SHR-CD: 75.6±10.8 vs. SHR-HFD: 4.48±17.4, and WKY-CD: 168.7±17.4 vs. WKY-HFD: 12.9±17.4, p<0.000001 (mean±SE, ANCOVA adjusted by HOMA). Analysis of liver Scd1 protein expression showed a particular pattern in the HFD groups, characterized by the presence of high levels of a monomeric protein band (32.2–36.6 Kda, p<0.003) and decreased levels of a dimeric protein band (61.9–66.1 Kda, p<0.02) regardless of the rat strain. Pharmacologic intervention with the peroxisome proliferator-activated receptor α agonist clofibrate reverted the liver phenotype and significantly modified the hepatic Scd1 transcriptional activity and protein expression.

**Conclusion:**

Diet-induced fatty liver is associated with the downregulation of hepatic *Scd1* transcript and de-dimerization of the protein, and these changes were not much affected by the status of peripheral IR.

## Introduction

Stearoyl-CoA desaturase (SCD), also known as fatty acid desaturase or delta (9)-desaturase, is a microsomal enzyme involved in the biosynthesis of monounsaturated fatty acids (MUFAs), primarily oleate (C18:1) and palmitoleate (C16:1). Therefore, SCD is a key enzyme in the regulation of the lipid metabolism and synthesis of triacylglycerols; SCD1 is differentially expressed in the liver.

Evidence from experimental and human studies has shown that SCD1 participates in the modulation of metabolic syndrome (MS)-associated phenotypes [1], including obesity [2], insulin resistance (IR) [3,4], and nonalcoholic fatty liver disease (NAFLD) [5,6], and it has been suggested that Scd1 is required for the onset of diet-induced hepatic IR in rodents [4].

Although SCD1 is a central lipogenic enzyme, its specific role in the development of human metabolic diseases is controversial. For example, previous *in vitro* studies have shown that SCD1 depletion leads to decreased synthesis of MUFAs and phospholipids and promotes the accumulation of saturated fatty acid-enriched unesterified fatty acids and triacylglycerol [7]. In addition, Scd1 inhibition in mice promotes atherosclerosis, reduces HDL cholesterol, and produces a significant enrichment of saturated free fatty acids, and the majority of these changes are originated in tissues involved in lipid biosynthesis, such as the liver [8].

Conversely, studies in mice with targeted disruption of Scd1 have suggested that the enzyme deficiency is associated with an activation of lipid oxidation in addition to reduced triglyceride synthesis and storage [9].

As previously mentioned, the metabolism of free fatty acids takes place primarily in the liver. Thus, the understanding of the behavior of hepatic SCD1 under metabolic or dietary stress is relevant to the understanding of the pathogenesis of the MS. Unfortunately, the modulation of liver *SCD1* transcriptional activity in NAFLD is still unclear. In fact, whereas some experimental studies have shown that hepatic Scd1 expression increases during high-fat diet (HFD) [10,11], other studies have indicated that the expression is significantly decreased [12]. An elegant review that summarizes the mechanisms behind the SCD1 activity and its relationship with metabolic disorders was recently published [13].

Also, there is still inconsistent data about the regulation of liver *SCD1* expression in human NAFLD. For instance, some clinical studies have shown that hepatic SCD1 activity increases with increasing liver fat content [14], being a determinant of liver fat accumulation under lipogenic dietary conditions [15] or obesity [16]. On the contrary, others have shown that SCD1 activity and mRNA expression are not upregulated in subjects with fatty liver and that the hepatic SCD1 activity index negatively correlates with hepatic fat content [16].

Interestingly, much of these changes observed in *SCD1* mRNA levels might be associated with the stability of the gene transcript, which is strongly affected by the levels of polyunsaturated fatty acids (PUFAs) [17,18]. In addition, PUFAs are able to repress the expression of the *Scd1* gene [19,20].

Another interesting feature is that the stability of the SCD proteins is much influenced by changes in the protein oligomerization and dimerization, which play an important role in regulating its half-life [21]. Indeed, the first reported that suggested that protein oligomerization play a role in the regulation of the stability of SCD enzymes was carried out in the human SCD2 (hSCD2) protein, which encodes a 37.5-kDa protein that shares 61% and 57% sequence identity with the human SCD1 and murine SCD2 enzymes, respectively [21]. Of note, both human SCD2 and rat SCD1 were proven to be oligomeric proteins in intact cells by transfection experiments[21].

Hence, to gain insights into the molecular events underlying the association between Scd1, fatty liver, and IR, we explored the hepatic expression of Scd1 in two experimental models of HFD-induced NAFLD: Sprague Dawley (SD) rats with increased visceral fat but without IR, and spontaneously hypertensive and IR rats (SHR), a rat model of genetic MS and cardiovascular disease (CVD), in comparison with its normotensive, insulin-sensitive control Wistar-Kyoto (WKY) strain. This strategy allowed us to explore the behavior of liver Scd1 expression in different metabolic environments.

## Materials and Methods

### Ethics Statement

All experiments with animals were performed according to the recommendations in the Guide for the Care and Use of Laboratory Animals of the “Comite Institucional del Cuidado y Uso de animals de Laboratorio (CICUAL) IDIM-UE” (Instituto de Investigaciones Medicas-Unidad Ejecutora); the CICUAL IDIM-UE approved this study. All animals received humane care, according to the Guide for the Care and Use of Laboratory Animals of Institute of Medical Research A Lanari-IDIM, University of Buenos Aires-National Council of Scientific and Technological Research (CONICET).

### Animal models

#### HFD-induced NAFLD without IR

Twelve-week-old male SD rats weighing 280±20 g were purchased from the Research Animal Facility of the School of Veterinary Medicine, University of Buenos Aires. After acclimatization for 1 week, the rats were randomly divided into two experimental groups. One group included 10 rats that received standard chow diet (CD) for 10 weeks (control group). The other group, including 15 animals, was allowed *ad libitum* access to HFD (40% w/w) consisting of bovine and porcine fat added to CD, as previously described [22], for the same period of time. The HFD provided 5340–5460 Kcal/kg, 13.8% of proteins and 26.4% of carbohydrates vs. 2900–3100 Kcal/kg, 23% of proteins and 44% of carbohydrates for the CD.

Fatty acid composition of the HFD is as follows: saturated free fatty acids: 42-43% (SFA=myristic (C14:0) + palmitic (C16:0) + stearic (C18:0), monounsaturated: 30-37% (MUFA=myristoleic (14:1) + palmitoleic (C16:1) + oleic (18:1), n-6 polyunsaturated fatty acids: 5-7% (n-6 PUFA=linoleic C18:2) + di-homo-gamma-linoleic C20:3 + arachidonic C20:4) + docosatetraenoic (C22:4) and n-3 PUFA 2-4% (linolenic C18:3 + eicospentaenoic C20:5 + doc-osapentaenoic C22:5 + docohexaenoic C22:6) (National Institute of Agricultural Research, INTA, La Pampa, Argentina).

#### HFD-induced NAFLD in a genetically determined model of IR and CVD

Sixteen-week-old male SHR (n=13) and WKY (n=14) rats (Charles River Laboratories, Wilmington, MA, USA) were included in this experiment. After acclimatization for 1 week, rats of both strains were randomly divided into two experimental groups. One group received CD for 10 weeks (control group, SHR: n= 6 and WKY: n=7). The other group was allowed *ad libitum* access to the same HFD described above for 10 weeks (SHR: n=7 and WKY: n=7).

In all the animals, housed under controlled conditions of temperature (23+1°C) and light (12-h light/dark cycle), food intake and body weight were monitored daily. At the completion of the study, food was withdrawn from 8:00 am to 4:00 pm before the animals were anesthetized with pentobarbital, and blood from individual rats was collected by cardiac puncture to determine the plasma and serum levels of different parameters. Liver tissue was excised and weighed, and intraperitoneal and retroperitoneal fat was weighted. The liver and fat weights were expressed as liver/rat length (taken from the nose to the tail origin) ratio (g/cm) to avoid the influence of body weight change. The liver was snap-frozen and stored at −76°C until gene expression analysis. A portion of each liver was fixed in 10% formalin for histologic analysis. Serum and sodium EDTA plasma was obtained by centrifugation and stored at −80°C until further use. Plasma insulin levels were determined with a commercial quantitative ultra-sensitive ELISA rat kit according to the manufacturer’s instruction (Crystal Chem Inc., Downers Grove, IL, USA). IR was calculated by the homeostasis model assessment (HOMA) index [fasting plasma insulin (µU/mL) × fasting plasma glucose (mmol/L)/22.5]. Leptin was measured by ELISA (Leptin ELISA Development Kit, PeproTech Inc., NJ, USA). All these measurements were done blinded to the experimental groups. At the end of each experiment, rats were sacrificed by intravenous administration of overdosed pentobarbital.

### Interventional experiment

Twelve-week-old male SD rats were given HFD for 8 weeks, after which they were randomly divided into two groups. For 4 weeks, along with the same access to HFD, one group received the agonist of the peroxisome proliferator-activated receptor α (Pparα) clofibrate (n=5 rats, 75 mg/kg intraperitoneally) every 24 h, and the other group (n=5 rats) was fed with HFD; an additional control group (6 rats) was fed with CD for 12 weeks. The animals were then killed, and liver tissue samples were obtained to measure the Scd1 mRNA and protein levels as previously described.

### Measurement of liver triglyceride content

The liver triglyceride content was determined with an automatic biochemical analytical system (Architect, Abbott, Buenos Aires, Argentina), and the results were expressed as micrograms of triglyceride per milligram of liver tissue (µg/mg liver).

#### Histologic analysis of liver tissue

With the use of light microscopy, the steatosis and necroinflammation levels from sections of formalin-fixed, paraffin-embedded samples stained with H&E and Masson’s trichrome were assessed. The degree of steatosis was assessed irrespective of the experimental groups and graded from 0 to 4+ according to the percentage of lipid-laden hepatocytes: 0, no steatosis; 1, fatty hepatocytes occupying less than 10% of the parenchyma; 2, between 10 and 30%; 3, between 30 and 60%; and 4, fatty hepatocytes occupying more than 60% of the parenchyma [23]. The severity of necroinflammatory activity was expressed on a 3-point scale, as follows: grade 1 (mild), grade 2 (moderate), and grade 3 (severe), as described by Brunt et al [23].

#### RNA preparation and real-time RT-PCR for quantitative assessment of mRNA expression

Total RNA was prepared from rat livers through the phenol extraction step method, with additional DNAse digestion. Total RNA was prepared from rat livers using the phenol extraction step method, with an additional DNAse digestion step. For RT-PCR, 3 µg of total RNA was reverse-transcribed using random hexamers and Moloney Murine Leukemia Virus (MMLV) reverse transcriptase (Promega, Wis, USA). Real-time PCR was performed for quantitative assessment of mRNA expression in an iCycler thermocycler (BioRad, Hercules, CA) using the fluorescent dye SYBR-Green (Invitrogen, Buenos Aires, Argentina). All the real-time PCR reactions were run in duplicate, and all the samples of the experimental groups were tested.

The relative abundance of the target gene mRNA was normalized to the amount of a housekeeping gene (TATA box binding protein, TBP) to carry out comparisons between the groups. TBP was found to be the most stable reference gene for testing liver mRNA expression among other housekeeping genes tested before starting the experiment [β-actin, peptidylprolyl isomerase A (cyclophilin A), and glyceraldehyde-3-phosphate dehydrogenase (Gapdh)]. The levels of mRNA were expressed as the ratio of the estimated amount of the target gene relative to the TBP mRNA levels with the use of the fluorescence threshold cycle values (Ct) calculated for each sample, and the estimated efficiency of the PCR for each product was expressed as the average of the sample efficiency values obtained [24]. The primer sequences are as follows: Scd1: forward 5’-CTGACCTGAAAGCTGAGAAG-3’ and reverse 5’-ACAGGCTGTGCAGGAAAGTT-3’; and TBP: forward 5’- TGGGATTGTACCACAGCTCCA-3’ and reverse 5’- CTCATGATGACTGCAGCAAAC C-3’.

#### Western blot analysis of liver Scd1 protein

Proteins from liver tissue were partially denatured in SDS sample buffer, separated with 8% SDS-acrylamide gel, and electrotransferred to Hybond-PVDF membranes (GE Healthcare UK Limited, Buckinghamshire, UK). After blocking with 5% nonfat dry milk in TBST buffer [20 mmol/L of Tris–HCl (pH 7.6), 137 mmol/L of NaCl, and 0.25% of Tween-20], the membranes were probed with rabbit polyclonal anti-Scd1 (Abnova, Taipei, Taiwan), followed by incubation with HRP-conjugated anti-rabbit polyclonal immunoglobulin G secondary antibody (GeneTex, Inc., GTX26795, Irvine, CA, USA). Equal protein loading (40 µg) was confirmed by re-blotting of the membranes with a goat polyclonal antibody against rabbit polyclonal anti-β-actin (1:500) (GeneTex, Inc., GTX16039, Irvine, CA, USA). Binding of the antibody was subsequently visualized with an enhanced chemiluminescence reagent (GE Healthcare UK Limited, Buckinghamshire, UK), and the band images were detected and analyzed with the LabWorks Analysis Software (Ultra-Violet Products Ltd, Cambridge, UK).

#### ELISA analysis of liver scd1 protein

Liver scd1 levels were measured in duplicate using a quantitative enzyme-linked immunoassay specific for rat according to the manufacturer’s instructions (USCN Life Science Inc, Houston, TX, USA); sensitivity: the minimum detectable dose of this kit is less than 1.38 pg/µL. All samples were tested blind to the experimental groups. Results are normalized by µg of liver protein in the extract.

#### Statistical Analysis

Quantitative data were expressed as mean ± SE. The data were also adjusted for body length whenever applicable. Pairwise mean differences were evaluated with the nonparametric Mann–Whitney test because most of the variables were ratios and not normally distributed, and/or nonhomogeneous variances between the groups were evident. For the comparison of more than two groups, we used the Kruskall–Wallis test. To test the differences in steatosis gradation (as a categorical response variable), we used ANCOVA with ordinal multinomial distribution, with probit as a link function and strain and diet as categorical factors adjusting for the indicated variables.

A value of p < 0.05 was considered to be statistically significant. We used the Statistica software package (StatSoft, Tulsa, OK, USA) for all the analyses.

## Experimental Results

**Figure 1:**
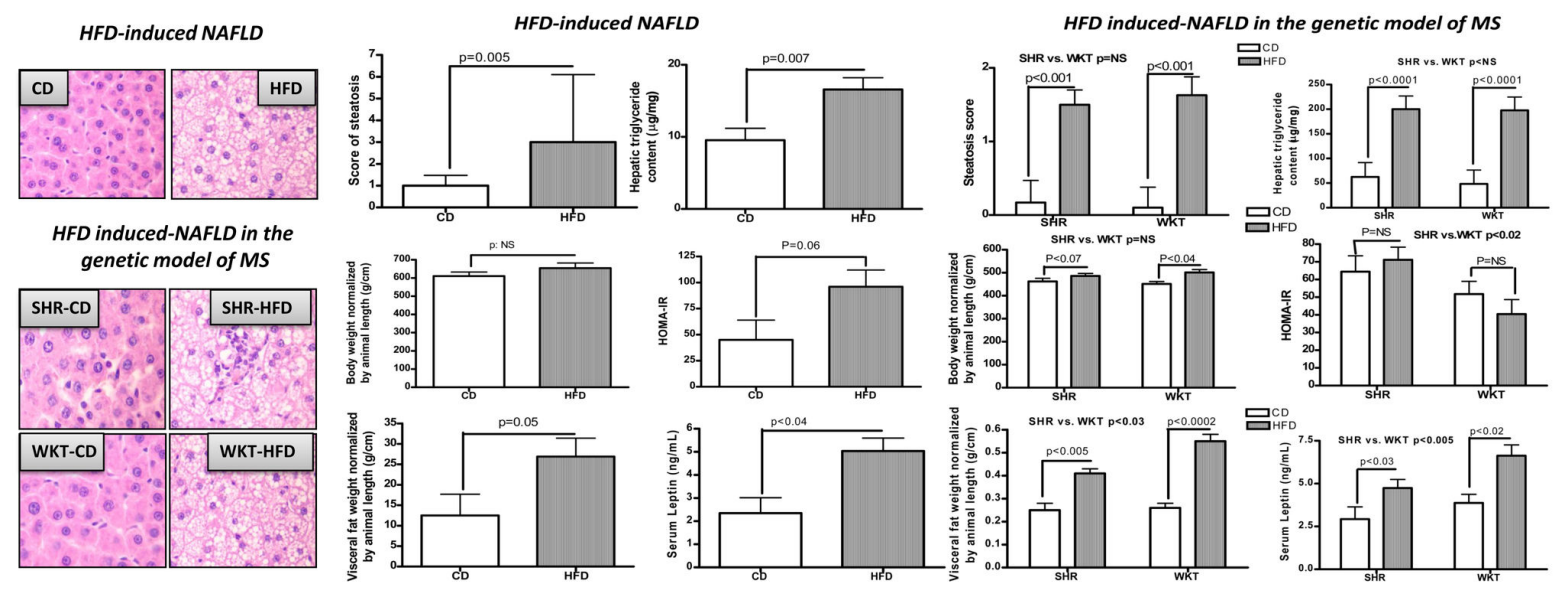
Left Panel: Liver histology of a representative animal from each experimental group. Hematoxylin and eosin staining of liver sections of a representative rat from each experimental group at the end of the experiment. The liver of rats fed with standard chow diet (CD) shows normal histology and absence of fat accumulation. The liver of rats fed with high fat diet (HFD) shows severe panlobular microvesicular and macrovesicular steatosis. Original magnification: 400×. Right panel: Phenotypic characteristics and biometric variables in the experimental models according to diet. Bar plots of steatosis score, liver triglyceride content, visceral fat weight (normalized by animal length), HOMA-IR, serum leptin levels (ng/mL), and body weight in Sprague Dawley (SD) rats that developed HFD-induced NAFLD without IR (upper panel) and in the HDF-induced NAFLD in the genetic model of MS in the SHR and its normotensive, insulin-sensitive control WKY strain (lower panel). HFD-induced NAFLD: Each bar represents the mean ± SE values of SD rats randomly divided into two experimental groups (CD: n=10; HFD: n=15). HFD-induced NAFLD in the genetic model of MS: Each bar represents the mean ± SE values of SHR (n=13) and WKY (n=14) randomly divided into two experimental groups (standard chow diet CD:SHR, n= 6 and WKY, n=7; high fat diet HFD: SHR, n=7 and WKY, n=7).

HFD-fed rats developed severe hepatic microvesicular and macrovesicular steatosis independently of the rat strain and the disease model (Figure 1, left panel). This finding was confirmed by biochemical analysis of the hepatic triglyceride content in rats of both experimental models (Figure 1, right panel).

In the model of HFD-induced NAFLD without IR, HFD-fed rats showed significant changes in body weight and visceral fat deposition when compared with their controls (Figure 1, right panel). Nevertheless, the plasma glucose in the HFD group was not significantly different from that in the CD group (199±6 and 186±7 mg/dL, respectively). Similarly, the plasma insulin levels and HOMA index, despite being lower in the CD group (96±35 and 45±19 µU/L, respectively), were not statistically different when compared with those observed in the HFD group (187±30 and 96±16 µU/L, respectively). Thus, despite the trend (p<0.06), HOMA-IR was not significantly different among rats consuming either CD or HFD (Figure 1, right panel).

On the other hand, in the genetic model of MS, exposure to HFD was associated with significant changes in body weight and visceral fat deposition in both the SHR and the control WKY strains (Figure 1). Nevertheless, only the SHR strains were insulin-resistant, as shown by significant differences in the HOMA index between strains (Figure 1, right panel).

**Figure 2:**
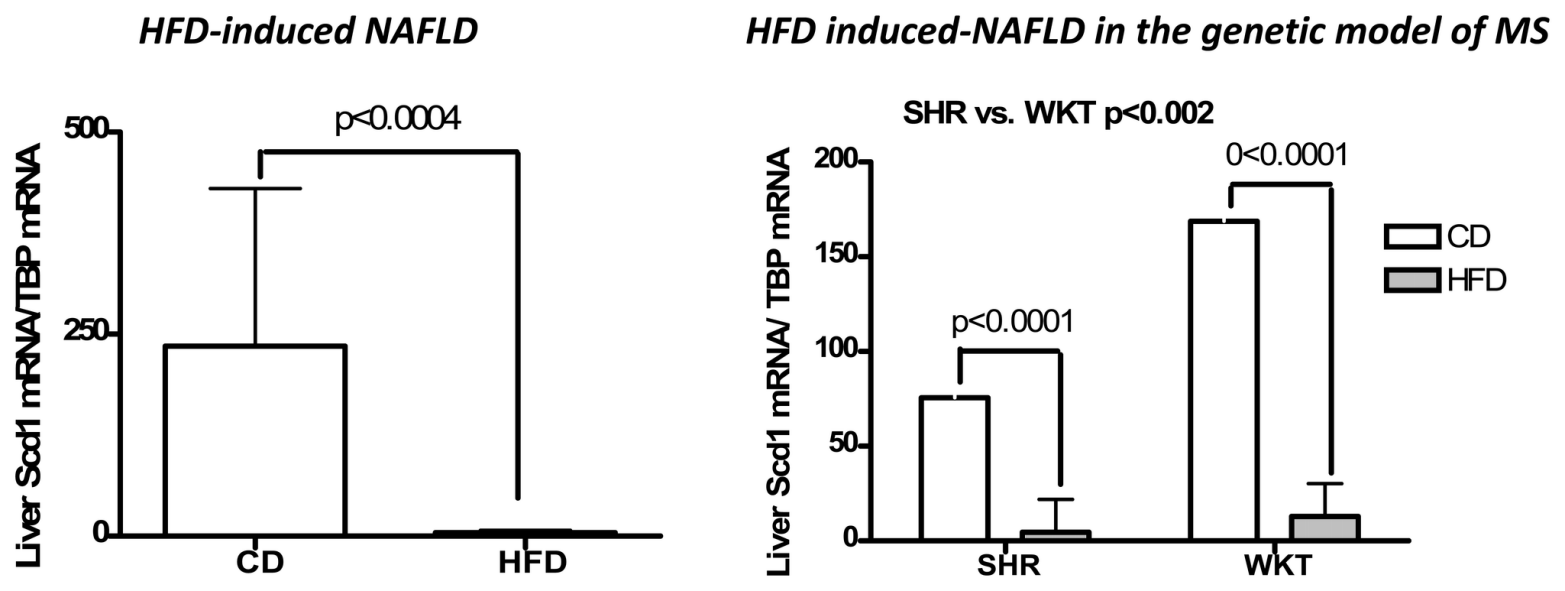
Analysis of liver *Scd1* mRNA abundance by quantitative real-time PCR in both experimental groups. Left panel: Each bar represents the mean ± SE values of Sprague Dawley (SD) rats randomly divided into two experimental groups (standard chow diet CD: n=10; high fat diet HFD: n=15). Right panel: Each bar represents the mean ± SE values of SHR (n=13) and WKY (n=14) divided into two experimental groups (CD:SHR, n= 6 and WKY, n=7; HFD: SHR, n=7 and WKY, n=7). The statistical significance of SHR vs. WKY indicates the comparison between strains independent of diet by two-way ANOVA. In each sample, the Scd1 expression was normalized by the expression of *Tbp* (TATA box binding protein).

The analysis of the liver mRNA expression of Scd1 showed substantial differences between rats fed with HFD vs. controls (Figure 2). In both experimental models, Scd1 mRNA significantly decreased when the rats were fed HFD. Nevertheless, changes in the hepatic Scd1 mRNA abundance were also observed in the insulin-resistant SHR vs. WKY rats even under CD.

**Figure 3:**
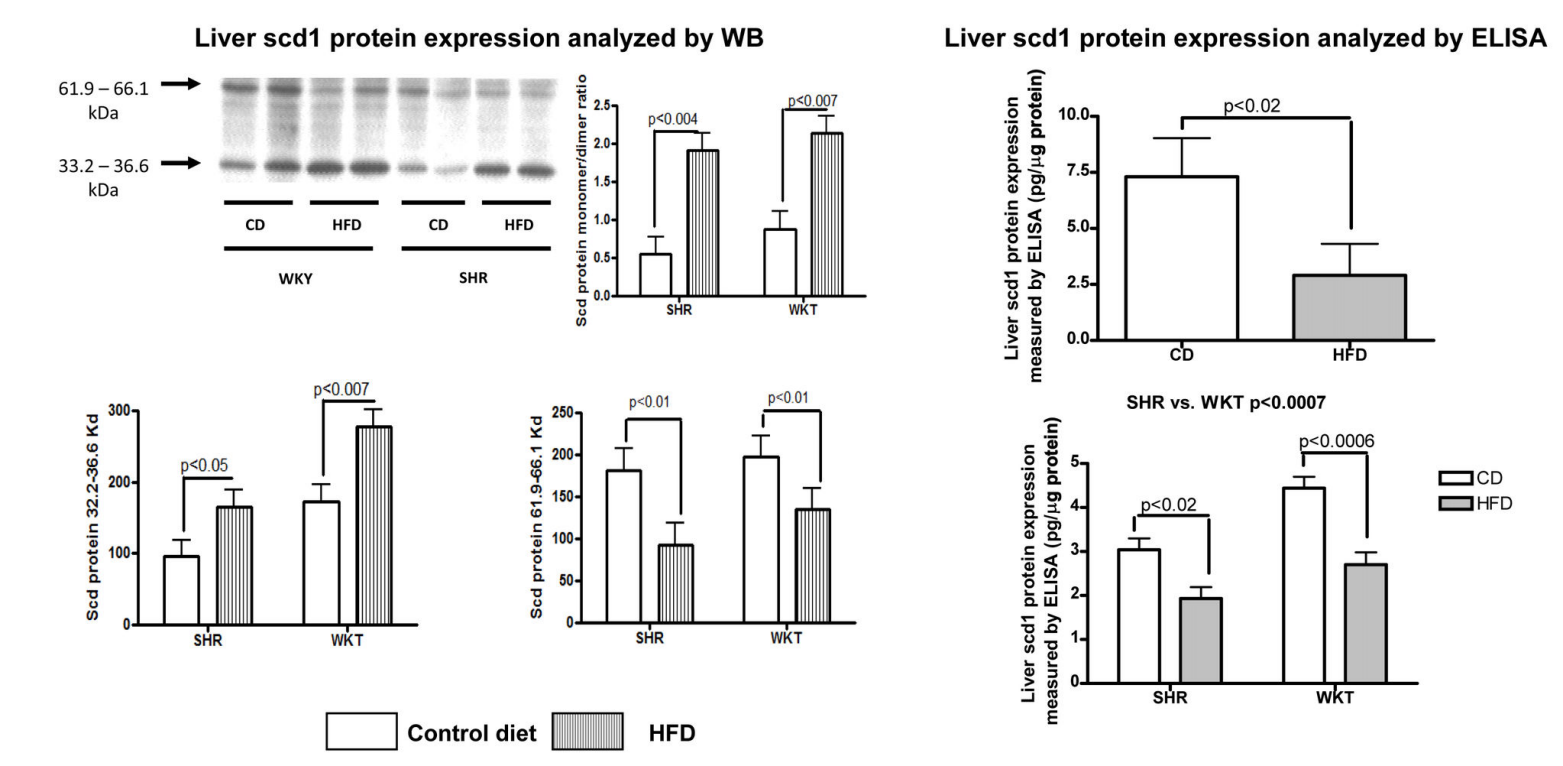
Analysis of liver Scd1 protein expression by Western blot and ELISA. Upper left panel: Representative analysis of liver Scd1 protein levels in each experimental model (SHR or WKY) with beta-actin as loading control. Upper right panel: Densitometric quantification of liver SCD1 monomer/dimer ratio protein levels. Values are means ± SE for 13 animals in the SHR group and 14 in the WKY group according to diet, as explained in the legends for Figures 1 and 2. Lower left and right panels: Densitometric quantification of the liver Scd1 monomeric and dimeric band, respectively, in each experimental group according to diet. Liver scd1 protein expression measured by ELISA. Upper right panel: HFD-induced NAFLD: Each bar represents the mean ± SE values of SD rats randomly divided into two experimental groups (CD: n=10; HFD: n=15). Lower right panel: Each bar represents the mean ± SE values of SHR and WKY divided into two experimental groups (CD:SHR, n= 6 and WKY, n=7; HFD: SHR, n=7 and WKY, n=7). Results are expressed pg/µl proteins.

To further explore whether changes in liver Scd1 mRNA were associated with changes in liver protein expression, we measured the liver abundance of Scd1 protein by Western blot and observed that HFD was associated with a decreased level of Scd1 transcript and loss of liver Scd1 protein dimerization (Figure 3). In fact, the analysis of liver Scd1 protein expression showed a particular pattern in the HFD groups, characterized by the presence of a high level of protein expression of a monomeric band (32.2–36.6 KDa, p<0.003) and a decreased level of a dimeric band (61.0–66.1 KDa, p<0.02) regardless of the rat strain (Figure 3). The monomer/dimer ratio was significantly (p<0.0002) higher in the HFD rats (HFD-SHR: 1.9 vs. CD-SHR: 0.55 and HFD-WKY: 2.1 vs. CD-WKY: 0.9). SHR showed decreased total Scd1 protein levels when compared with WKY rats (p<0.02).

Liver tissue levels of scd1 protein were also measured by ELISA and we observed that HFD was significantly associated with decreased protein levels in both experimental rat models (Figure 3, right panels).

### Interventional experiment

Because previous data have shown that hepatic SCD1 gene expression is regulated by peroxisome proliferators (PPARs) and that clofibrate induces liver *SCD1* mRNA levels up to 22-fold in 30 h [25], we decided to explore the effect of this PPAR alpha agonist on hepatic Scd1 gene and protein expression. Previous results have shown that Scd1 mRNA and protein exhibited similar behavior in both experimental models; hence, we chose one of them, as explained in the Methods section, to avoid the unnecessary use of animals.

**Figure 4:**
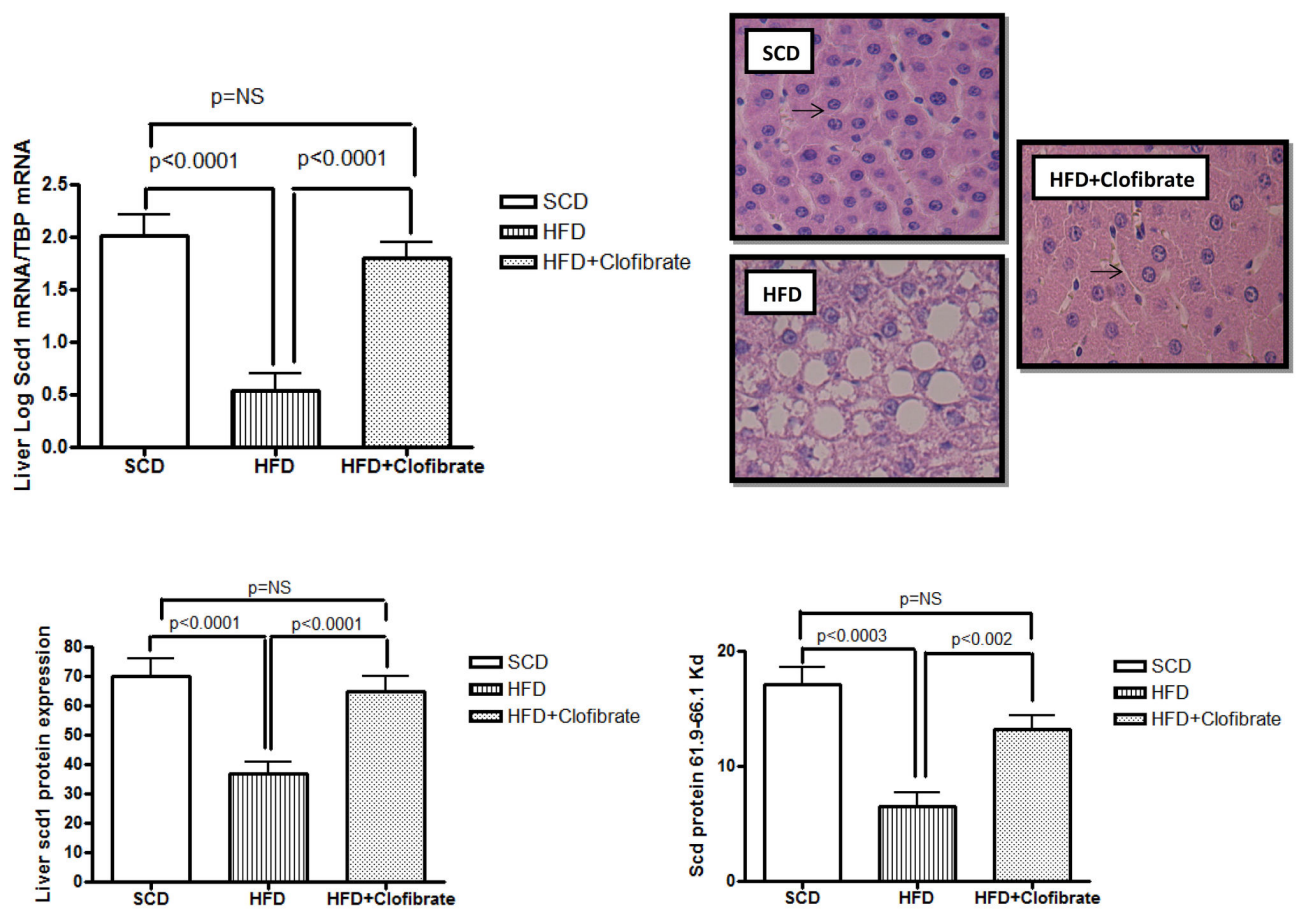
Interventional experiment: Evaluation of the effect of clofibrate, an agonist of PPAR alpha, on hepatic Scd1 mRNA, protein expression and liver histology. Each bar represents the mean ± SE values of Sprague Dawley (SD) rats fed with standard chow diet CD (n=6), high fat diet HFD (n=5), and HFD plus clofibrate (n=5). In each sample, the gene expression was normalized to the expression of *Tbp* (TATA box binding protein). The arrow shows the characteristic hypertrophy of hepatocytes associated with PPAR alpha induction. The liver histology of a representative animal from each experimental group shows the hematoxylin-and-eosin staining of liver sections at the end of the experiment. Original magnification: 400×.

Interestingly, clofibrate was found to not only improve fatty liver but also modify the effects of HFD on Scd1 gene and protein expression (Figure 4) because we observed that liver Scd1 mRNA and protein expression were significantly induced by clofibrate when compared with that in CD-fed animals (Figure 4).

## Discussion

In this study, we observed that diet-induced NAFLD is associated with a dramatic decrease in Scd1-mRNA abundance, which was not affected by the status of peripheral IR. Indeed, HFD produced a significant downregulation of liver Scd1 mRNA in both experimental models; this was also associated with changes in Scd1 protein expression, which behaved in a similar fashion. Thus, whereas the modulation of Scd1 mRNA induction and protein content is part of a robust system that is highly sensitive to dietary and hormonal factors, at least in the liver tissue, it is not critically modified by the status of IR. Moreover, we observed that HFD-induced NAFLD was associated with significant changes in Scd1 protein dimerization, which was reverted by the use of clofibrate, a powerful PPAR alpha agonist that was also able to improve fat accumulation in hepatocytes.

Previous evidence showed that the level of PUFAs strongly influence the abundance of *Scd1*-mRNA in the liver. For instance, Landschulz et al. showed that the levels of primarily polyunsaturated (18:2 and 18:3) fat markedly suppress the expression of the hepatic *SCD1* message [26]; a similar observation was replicated recently by McNamara et al [27].

Furthermore, it is worth mentioning an additional comment regarding the putative constitutive differences in liver *Scd1* mRNA expression between SHR and WYT. Indeed, there is scarce information about this issue; nevertheless a previous work reported that a SHR/NIH corpulent rat substrain (SHR/NDcp), which was obtained by crossing SHR/NIH corpulent rat with SHR, shows 5.5 times higher *scd1* gene expression than that of WKY but also showed a tendency of the original SHR strain to have decreased levels of *Scd1* expression in comparison with WKY rats [28].

To our knowledge, an association between changes in liver Scd1 protein expression and changes in the pattern of protein dimerization in relation with NAFLD has not been previously reported.

Protein oligomerization and dimerization affects protein function, cross-talk with other proteins, assembly with protein complexes involved in gene expression, protein structure, and protein relationship with cellular organelles [29]. Accordingly, Zhang et al. showed *in vitro* that the dimerization and oligomerization of SCD proteins play an important role in regulating the half-life of SCD enzymes [21]. In addition, they showed that although SCD oligomers are not stable, they do not affect protein expression because degradation products increase proportionally with the SCD protein levels [21]. This novel mechanism of SCD protein regulation might significantly affect the enzyme activity and function. These observations makes a lack of specificity of the antibody we used although possible, unlikely. Considering the leading role of hepatic lipid metabolism in the pathogenesis of MS [30–32], it is tempting to speculate that changes in the *SCD1* expression in the liver might significantly alter the hepatic lipid metabolism aggravating the systemic metabolic derangement observed in patients with MS.

In fact, despite the heterogeneous published evidence about the impact of the hepatic *SCD1* transcriptional activity on liver and systemic lipid metabolism, it is evident that decreased *SCD1* activity is associated with lipoapoptosis [33], lipotoxicity [6], and inflammation [8,33,34].

**Figure 5:**
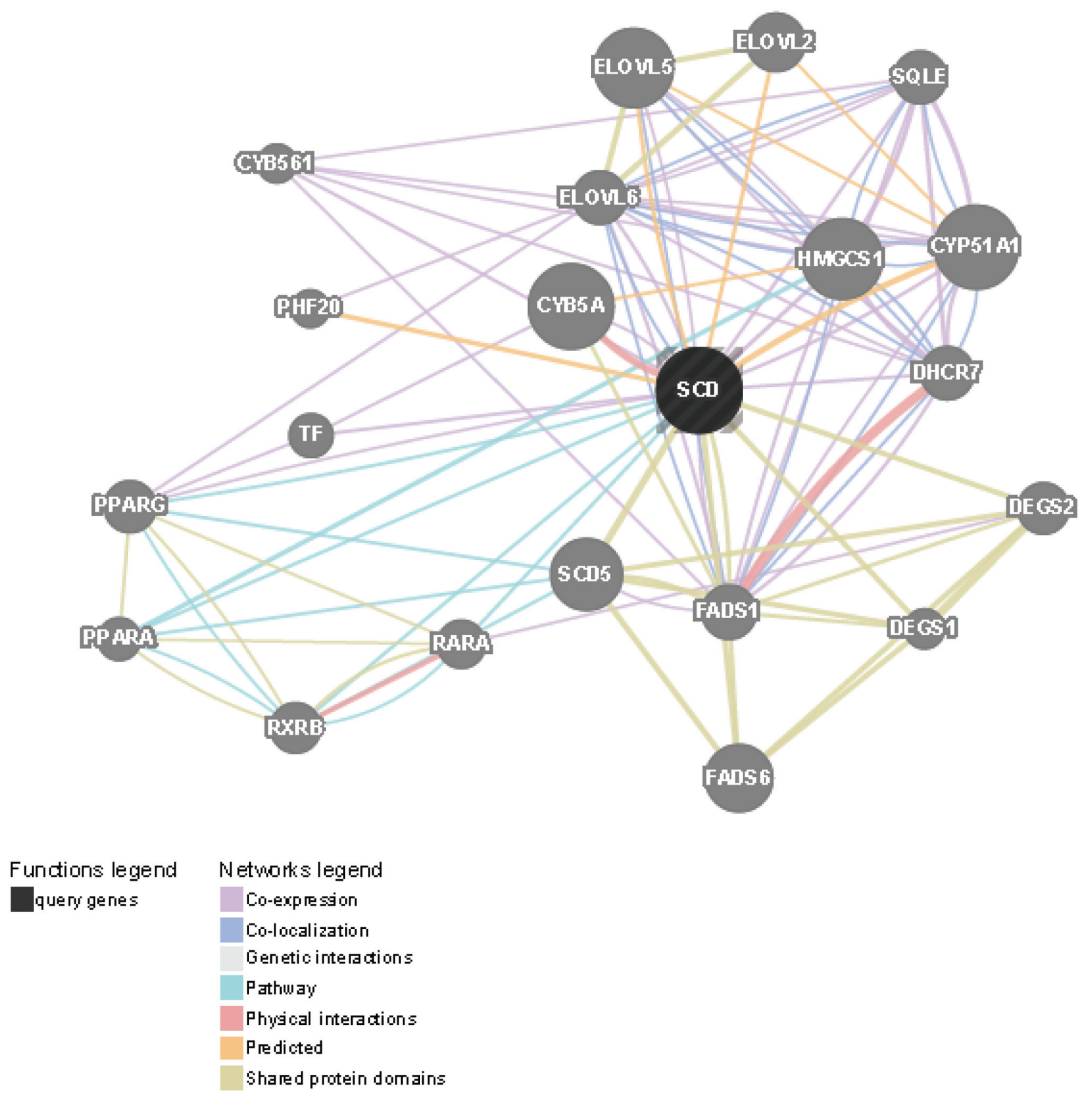
Results of functional association analysis by the bioinformatics resource GenMANIA (available at http://www.genemania.org/, Toronto, Canada). SCD (SCD1): stearoyl-CoA desaturase (delta-9-desaturase); CYB5A: cytochrome b5 type A (microsomal); CYP51A1: cytochrome P450, family 51, subfamily A, polypeptide 1, HMGCS1 3-hydroxy-3-methylglutaryl-CoA synthase 1 (soluble); ELOVL5: ELOVL fatty acid elongase 5; SCD5: stearoyl-CoA desaturase 5; FADS6: fatty acid desaturase domain family, member 6; ELOVL2: ELOVL fatty acid elongase 2; FADS1: fatty acid desaturase 1; ELOVL6: ELOVL fatty acid elongase 6; DHCR7: 7-dehydrocholesterol reductase; SQLE: squalene epoxidase; PPARG: peroxisome proliferator-activated receptor gamma; DEGS2: degenerative spermatocyte homolog 2, lipid desaturase; RXRB: retinoid X receptor, beta; RARA: retinoic acid receptor, alpha; TF: transferring; PPARA: peroxisome proliferator-activated receptor alpha; DEGS1: degenerative spermatocyte homolog 1, lipid desaturase; CYB561: cytochrome b-561; PHF20: PHD finger protein 20.

**Figure 6:**
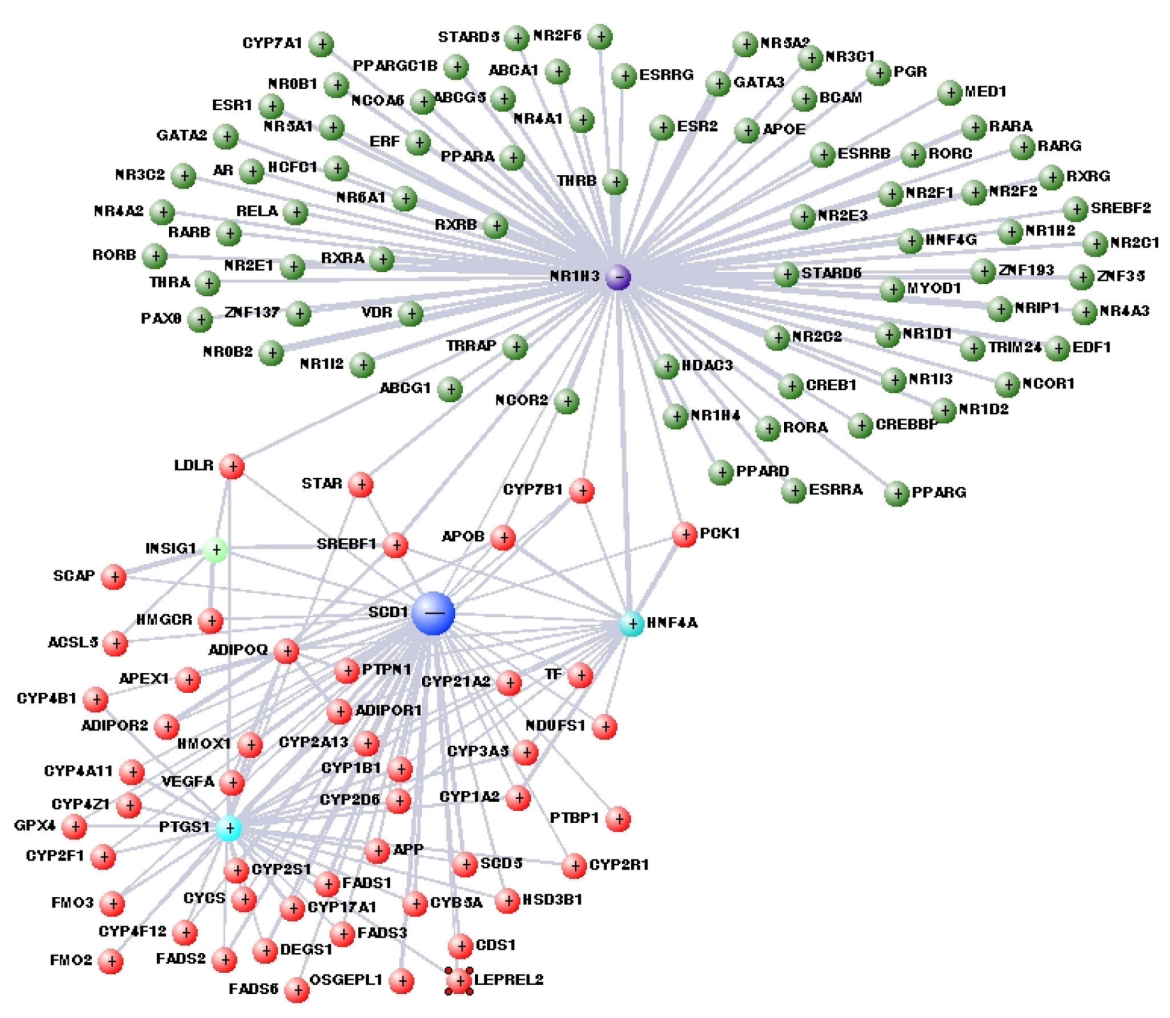
Analysis of interaction pathways (http://visant.bu.edu/) of SCD1; the metagraph structure allows the visualization of bionetworks constructed by integrative data-mining features, which permits a large number of functional associations for 103 different species. Direct SCD1-related nodes are shown in red. A few nodes are labeled for the sake of simplicity. The gene labels are official names.

Functional association analysis with the use of the bioinformatic resource GenMANIA [35] depicts the SCD1 genetic interactions, related pathways, and protein co-expression, co-localization, and domain similarity (Figure 5). Particularly, the analysis shows shared molecular pathways between PPAR alpha and gamma and SCD1, including pathways related to sequestering triglycerides (p< 0.04), as well as negative regulation of lipid (p<0.05) and cholesterol storage (p<0.045). More interestingly, the common molecular pathways among PPAR alpha, PPAR gamma, and SCD1 also include negative regulation of macrophage-derived foam cell differentiation (p <0.045), an observation that illustrates the involvement of the downregulation of hepatic *SCD1* transcription in the worsening of the fatty liver phenotype [6]. A more detailed analysis and modeling of the biological networks focused on SCD1 is shown in Figure 6. The additional metanodes containing genes annotated under NR1H3 or retinoid X receptor (RXR) (green nodes in circle), and metanodes around HNF4a, INSIG1, and PTGS1 are noteworthy.

Finally, we observed that interventional pharmacologic approaches, such as improving NAFLD by clofibrate, not only reverted the liver phenotype but also significantly affected the hepatic Scd1 transcriptional activity and protein dimerization. Thus, modulation of the hepatic SCD1 expression in NAFLD might directly affect the global lipid metabolism. Further investigations on humans must be carried out to precisely define the role of hepatic SCD1 protein dimerization on enzymatic activity and systemic lipid metabolism.
